# Integrating Machine Learning-Based Virtual Screening With Multiple Protein Structures and Bio-Assay Evaluation for Discovery of Novel GSK3β Inhibitors

**DOI:** 10.3389/fphar.2020.566058

**Published:** 2020-09-11

**Authors:** Jingyu Zhu, Yuanqing Wu, Man Wang, Kan Li, Lei Xu, Yun Chen, Yanfei Cai, Jian Jin

**Affiliations:** ^1^ School of Pharmaceutical Sciences, Jiangnan University, Wuxi, China; ^2^ Jiangsu Institute of Hematology, National Clinical Research Center for Hematologic Diseases, NHC Key Laboratory of Thrombosis and Hemostasis, The First Affiliated Hospital of Soochow University, Suzhou, China; ^3^ School of Electrical and Information Engineering, Institute of Bioinformatics and Medical Engineering, Jiangsu University of Technology, Changzhou, China

**Keywords:** glycogen synthase kinase-3 beta inhibitor, GSK3β, virtual screening, molecular docking, pharmacophore, naive Bayesian classification, molecular dynamics simulation

## Abstract

Glycogen synthase kinase-3β (GSK3β) is associated with various key biological processes, and it has been considered as a critical therapeutic target for the treatment of many diseases. However, it is a big challenge to develop ATP-competition GSK3β inhibitors because of the high sequence homology with other kinases. In this work, a novel parallel virtual screening strategy based on multiple GSK3β protein structures, integrating molecular docking, complex-based pharmacophore, and naive Bayesian classification, was developed to screen a large chemical database, the 50 compounds with top-scores then underwent a luminescent kinase assay, which led to the discovery of two GSK3β inhibitor hits. The high screening enrichment rate indicates the reliability and practicability of the integrated protocol. Finally, molecular docking and molecular dynamics simulation were employed to investigate the binding modes of the GSK3β inhibitors, and some “hot residues” critical to GSK3β affinity were highlighted. The present study may provide some valuable guidance for the development of novel GSK3β inhibitors.

## Introduction

Glycogen synthase kinase-3 (GSK3) is an evolutionarily very conserved serine/threonine kinase that is ubiquitous in mammalian eukaryotic cells, and it presents a broad spectrum of cellular functions, like cell division, differentiation, transcription, apoptosis, and so on (Frame and Cohen, 2001; Hu et al., 2018). GSK3 contains two functionally distinct isoforms, α and β, and these two isoforms share a 98% sequence identity within the active domain (MacAulay and Woodgett, 2008). Among them, GSK3β has received more attention because of its central role in the regulation of many important metabolic and signaling proteins, structural proteins and transcription factors, that depicts GSK3β as a promising target for the treatment of cancer, neurodegenerative diseases, neuropsychiatric diseases, and other major diseases (Cohen and Frame, 2001; Osolodkin et al., 2013; O’Leary and Nolan, 2015; Maqbool and Hoda, 2017; Abdul et al., 2018). Therefore, a certain number of GSK3β inhibitors with different kinds have been discovered in last decades (Wagman et al., 2004; Maqbool and Hoda, 2017), and some have been pushed into the clinical trials (Wu et al., 2019). GSK3β inhibitors can be roughly classified into two categories: non-ATP competition and ATP competition inhibitors (Force and Woodgett, 2009; Swinney et al., 2016; Dey et al., 2017; Saura et al., 2017). These two types of inhibitors represent two different focuses on the development of GSK3β inhibitors: affinity and selectivity. non-ATP inhibitors binding to the ATP outside area usually contain higher selectivity but lower affinity to GSK3β, but ATP inhibitors acting to ATP pocket are just the opposite. Nowadays, a large number of ATP competition GSK3β inhibitors have been developed, but no selective GSK3β inhibitor has been FDA approved so far (Sahin et al., 2019). As mentioned above, GSK3β show a 98% sequence identity to GSK3α, besides, GSK3β share a highly conserved sequence in many other kinases, such as cyclin-dependent kinases (CDK) (Bax et al., 2001), that makes it challenging to develop selective GSK3β inhibitors. Therefore, the application of appropriate methods for developing high selective GSK3β inhibitors has become critical and urgent.

**Figure 1:**
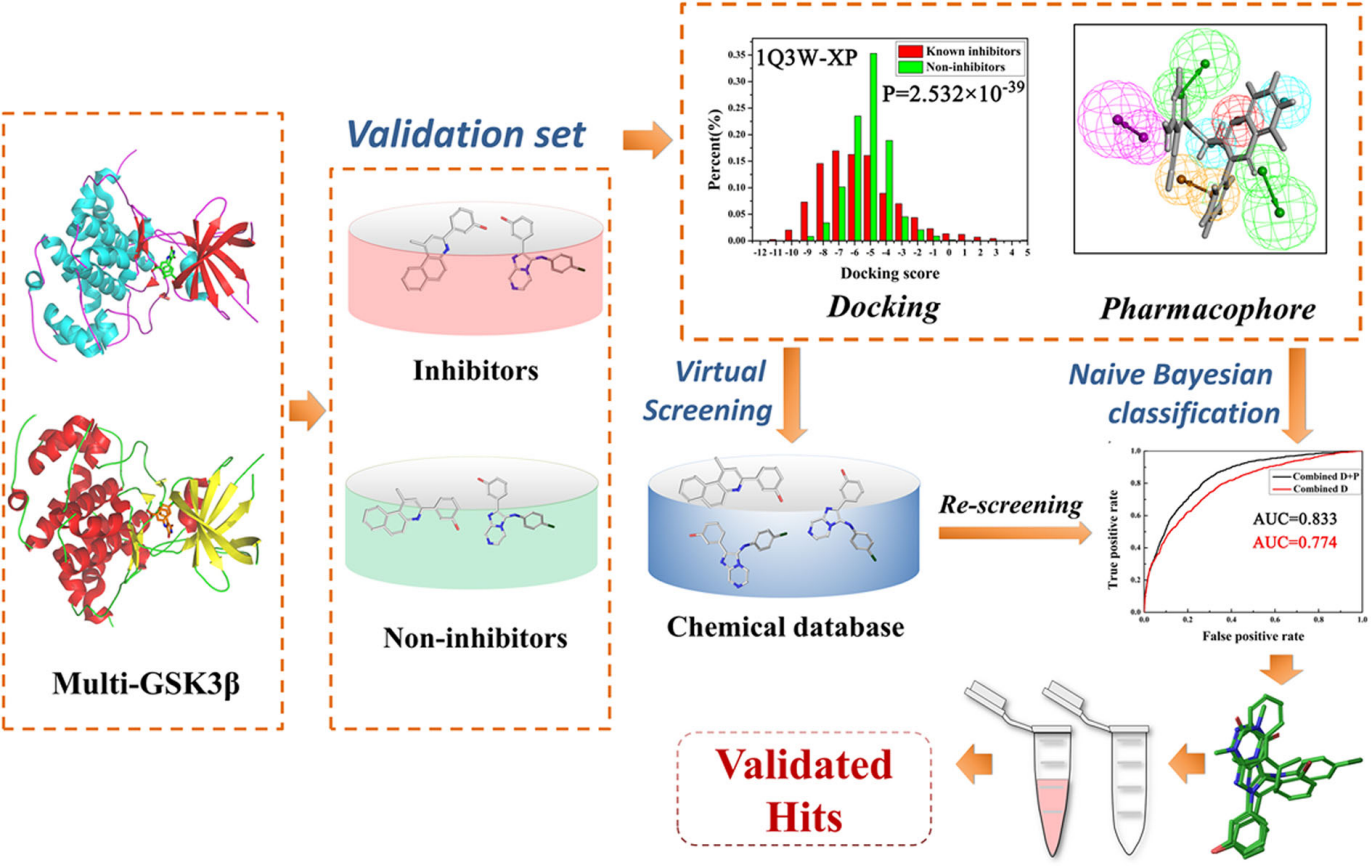
The workflow of this study.

As is known to all, traditional lead kinase inhibitors discovery based on experimental systems is an expensive, inefficient and lengthy process because of its screening against a broad panel of diverse kinases. As a counterpart to experimental high-throughput screening, virtual screening (VS) is able to virtually screen large compound databases and has become a good choice for the discovery of novel inhibitors (Hou and Xu, 2004; Amaro and Li, 2010). VS based on molecular docking has received more and more attention (Tanrikulu et al., 2013). For conventional VS, only one protein structure is used in most docking programs, and the protein almost performs the “rigid” structure to maintain optimum efficiency of VS. Thus, integrated strategies for VS in a parallel manner may be the most appropriate to balance the efficiency and precision of VS, and some successful cases in the field of kinase inhibitor development have been reported (Bajorath, 2002; Holliday et al., 2011; Tian et al., 2014; Fan and Huang, 2017; Zhou et al., 2018). Among them, naive Bayesian analysis, a machine learning algorithm, has been widely applied to drug discovery processes such as high-throughput VS, ADMET (absorption, distribution, metabolism, excretion, and toxicity) properties evaluation, and SAR (structure-activity relationship) analysis (Liu, 2004; Chen et al., 2011; Ekins et al., 2019). Bayesian analysis is a well-known statistical algorithm and it could scale linearly with the number of samples comparing with traditional fitting methodologies, thus, Bayesian analysis could be fast and easily automated to process large amounts of data (Rogers et al., 2005; Tian et al., 2013a). Moreover, the Bayesian model focuses on the more important features of samples, and then assigns greater weight to the characteristics to distinguish the “good” samples from the large amounts of sample, that would significantly lead to a higher quality enrichment (Klon et al., 2004; Rogers et al., 2005). In this study, we present an efficient and reliable predictive strategy for parallel Bayesian machine learning-VS integrating molecular docking and complex-based pharmacophore based on multiple GSK3β proteins (the workflow is illustrated in **Figure 1**), 50 potential inhibitors of GSK3β were purchased and some compounds with potent GSK3β inhibitory activity were confirmed by a series of biochemical studies. Finally, the GSK3β binding mechanisms of these inhibitors were well analyzed through molecular docking and molecular dynamics simulation. It could provide some important guidance for the discovery of promising GSK3β inhibitors from a huge chemical database for the treatment of related diseases.

## Methods and Materials

### Preparation of GSK3β Complexes and Validation Dataset

A total of 54 crystallographic structures of GSK3β complexes were retrieved from the RCSB Protein Data Bank (PDB) (Berman et al., 2000). Firstly, the residues within 10 Å of each apo-ligand were reserved and structurally aligned with STAMP algorithm in VMD (**Figure S1**) (Humphrey et al., 1996; Tian et al., 2014). Secondly, the root-mean-square deviation (RMSD) values from the alignment were used to create a phylogenetic tree through the *phylogenetic tree* module of VMD (**Figure S2**). And then, these complexes could be classified eight types according to the differences of the GSK3β structures. Finally, eight representative complexes with the highest resolution within each type were chosen, namely, 1Q3W (Bertrand et al., 2003), 1Q4L (Bertrand et al., 2003), 2OW3 (Zhang et al., 2007), 3L1S (Arnost et al., 2010), 4B7T (Tahtouh et al., 2012), 4J71, 4NM3 (Stamos et al., 2014) and 4PTG (Sivaprakasam et al., 2015) (highlighted in red in **Figure S2**).

In order to evaluate the “screening power” of these eight protein structures, a validation dataset was built, including the known GSK3β inhibitors and non-inhibitors. The non-duplicated GSK3β inhibitors with definite biological activity were obtained from the BindingDB database (Liu et al., 2007), and the non-inhibitors were randomly selected from the ChemDiv database through the *Find Diverse Molecules* module in Discovery Studio 3.5 (DS3.5), and the ratio of non-inhibitors versus inhibitors was set to 1:20. At last, 800 inhibitors and 16,000 non-inhibitors were chosen for further study.

### Molecular Docking Procedure

The crystallographic structures of eight GSK3β complexes were employed as initial receptors for VS. Molecular docking simulations were carried on by the *Glide* module in Schrodinger (Friesner et al., 2006). First, each complex was prepared using the *Protein Preparation Wizard* in Schrodinger, to remove crystallographic water molecules, add hydrogen atoms, assign protonated states, assign partial charges. Afterward, each complex was minimized with the OPLS-2005 force field until the RMSD reached a maximum value of 0.3 Å. The compounds in the validation set were processed through the *LigPrep* protocol in Schrodinger with the default parameters set. Finally, a bounding box of size 10 × 10 × 10 Å was generated with the co-crystallized ligand as centroid using the *Receptor Grid Generation* module for each system.

Subsequently, the “scoring power” was evaluated to estimate the docking precision of these eight systems. The crystallized ligand was first extracted from each GSK3β complex, and then re-docked into the corresponding binding site, the RMSD between the docking pose and crystallographic conformation of the inhibitors was calculated. On the other hand, the “screening power” was also investigated. All molecules in the validation dataset were docked into the binding site of each protein with the Standard Precision (SP) or Extra Precision (XP) scoring modes. The student ’s t-test was used to measure the difference in the distribution of docking scores between active inhibitor and non-inhibitors.

### Pharmacophore Procedure

The eight crystal GSK3β structures were also used as the initial receptors for complex-based pharmacophore models. First, the proteins were prepared through the *prepare protein* module in DS3.5 to add polar hydrogen, remove water molecules, repair the broken chain, and add the *CHARMm* field with the default parameters. Second, all molecules in the validation set were prepared through the *prepare ligands* module in DS3.5. Then, the *Receptor-Ligand Pharmacophore Generation* (RLPG) module was employed to generate the complex-based pharmacophore models for eight GSK3β complexes (Hou et al., 2012). A set of pharmacophoric features were identified from each system (Sutter et al., 2011), including hydrogen bond acceptor (HBA), hydrogen bond donor (HBD), hydrophobic (HYD), negative ionizable (NI), positive ionizable (PI), and ring aromatic (RA) features of ligands. And the parameters were kept as the default setting except for the minimum number of the pharmacophore features was set to 3.

The selectivity of the pharmacophore model is evaluated through genetic function approximation (GFA) scoring function (Rogers and Hopfinger, 1994; Meslamani et al., 2012). For each complex structure, the generated models are ranked according to their selectivity scores evaluated by the GFA model, and thus 10 top-ranked pharmacophore models would be produced, and feature(S) was defined as the pharmacophore features with the highest selectivity. Besides, in order to investigate the capability of each model to distinguish between inhibitors and non-inhibitors, a validation set containing 800 active GSK3β inhibitors and 16,000 non-inhibitors mentioned above was constructed. The pharmacophore features of the pharmacophore model with the best discrimination between inhibitors and non-inhibitors were set to feature(D), and the area under receiver operating characteristic (ROC) curve (AUC) was calculated to evaluate this discrimination capability. Moreover, the molecules in the validation set were all mapped onto the pharmacophore models to generate the fit values.

### Naive Bayesian Classification

Consequently, naive Bayesian classification (NBC) based on multiple protein structures was employed to evaluate the screening accuracy. This machine learning approach has been proved to significantly increase the hit rate of virtual screening (Tian et al., 2013b; Li et al., 2014). First, the data matrix consists of above docking scores (from molecular docking) and fit values (from pharmacophore) were used as the independent variables (X); and 1/0 was used as the response variable (Y), in which 1 presents inhibitor and 0 means non-inhibitor. Then, NBC was developed using the *Create Bayesian Model* module in DS3.5 (Hou et al., 2012) to distinguish the inhibitors from non-inhibitors. The prediction precision of each classifier was evaluated by the AUC value for discriminating the GSK3β inhibitors from non-inhibitors. Finally, the best Bayesian classifier was utilized to re-score the compounds.

### Integrated Virtual Screening

The docking-VS was performed to screen the ChemDiv database (approximately 2,000,000 compounds). First, each compound was docked into the binding pockets of eight GSK3β with corresponding scoring modes. Then, 10,000 compounds with the best docking scores were obtained and then mapped onto the pharmacophore models generated from the GSK3β complexes. Afterward, 2,000 compounds with the best fit values were chosen and then to re-screening with the best Bayesian classifier. Last, the 100 compounds with top-ranked by the Bayesian scores were assessed the drug-likeness by *Filter by Lipinski and Veber Rules* module in DS3.5. Finally, 50 compounds were purchased from ChemDiv for subsequent GSK3β inhibitory assay *in vitro*.

### Kinase Assay

GSK3β kinase activity was measured by ADP-Glo Kinase Assay system from Promega (Product code V1991) (Auld et al., 2009; Zegzouti et al., 2009; Cho et al., 2011), which is a non-radioisotope, homogeneous, ADP quantitative kit based on a luminescent kinase assay. Compounds were initially tested in triplicate at 20 µM, and then, those molecules which contain an inhibitory activity greater than 50% were chosen to further test in a nine-point dose curve with 2-fold serial dilution starting from 40 µM (three times). The assay protocol was listed as below: GSK3β protein was firstly incubated with 1μg/ml of the peptide substrate, second, the test compounds were added into the solution for 60 min at 25°C with a GSK3β reaction buffer, which contained 40 mM Tris (pH = 7.5), 20 mM MgCl_2_, 0.1mg/ml BSA, 50 μM DTT, and 50 μM ATP. The assays were done in 384-well white plates with a total reaction volume of 5 μl per well. Then, the reactions were terminated through the introduction of 5 μl ADP-Glo reagent assay, and the assay plate was incubated for 40 min. Finally, 10 μl of kinase detection buffer was added to convert ADP to ATP and introduce luciferase and luciferin to detect ATP.

### Promiscuity Assessment

The promiscuity of Cpd49 was evaluated by an online program, Badapple (http://pasilla.health.unm.edu/tomcat/badapple/badapple). the simplified molecular input line entry specification (SMILES) formats of Cpd49 was entered into the input box and the results would be generated automatically.

### Molecular Docking and MD

The 3D structure of compound22 (Cpd22) and Cpd49 were sketched using maestro, LY2090314 was retrieved from the PubChem Compound database. All these inhibitors were prepared by the *Ligprep* module with the OPLS-2005 force field in Schrodinger. The crystal structure of GSK3β with the highest resolution, 1Q4L, and the crystal structure of GSK3α, 2DFM (Wang et al., 2019), were prepared as the initial receptors for the molecular docking through the *Protein Preparation Wizard* module. The grid file of the GSK3β protein was generated following the step in *Chapter 2.2*, and that of GSK3α was generated with the specified residues, Val101, Pro102, and Arg107 (hinge region), as centroids. Then Cpd22, Cpd49, and LY2090314 were respectively docked into the GSK3β protein, and Cpd49 was docked into the GSK3α protein using the *Glide* module (XP mode), and the best-docked structure of Cpd49 in the GSK3β-ligand system was chosen for the following MD simulation.

The Cpd49 with the best bioactivity was conducted to MD with *Desmond* package in Schrodinger (Chapon, 2014), and the docked GSK3β/Cpd49 complex was chosen as the initial system. All amino acid interactions in the protein were modeled with the OPLS-2005 force field. The simulation system was solvated in a 10 Å orthorhombic box with periodic boundary condition, and built with the 3-Point Transferable Inter-molecular Potential (TIP3P) water model. Afterward, the whole system was neutralized with salt concentrations of 0.15 M of Na^+^ ions. Before MD simulation, the system was minimized with OPLS-2005 force field. And then, the 200-ns MD was performed using the NPT ensemble under a target temperature of 310 K and a target pressure of 1 atm, the energy and atomic coordinate trajectory recording interval were set to 20 ps. Thereafter, the RMSD and protein-ligand contacts were all calculated with the *Simulation Interaction Diagram* protocol in *Desmond* package.

### Results and Discussion

#### Validation of Docking-Based Virtual Screening

To investigate the prediction capability and reliability of molecular docking, the screening ability, including “scoring power” and “screening power”, was evaluated. Scoring power shows the reliability of the docking program, namely, whether it can predict the real binding conformation between inhibitors and proteins (Shen et al., 2020). Herein, the RMSD between docking pose and crystallographic structures was calculated to reveal this “power” and RMSD ≤ 2.0 Å was used as a criterion. Firstly, the original ligands of eight GSK3β complexes were extracted and then re-docked into the corresponding binding pocket of the protein. The RMSD values were calculated and the values were summarized in **Table 1**. Generally, the docking program, whether SP mode or XP mode, basically reproduces the experimental conformation (all RMSD values ≤ 2.0 Å), indicating the eight chosen protein structures all satisfy the docking accuracy with *Glide* module.

**Table 1:** Scoring power and screening power of the eight GSK3β protein structures.

Complex	P-value		RMSD (Å)	
	SP	XP	SP	XP
**1Q3W**	2.079×10^−23^	**2.532×10** ^−^ **^39^**	0.72	0.58
**1Q4L**	0.016	**6.899×10** ^−^ **^36^**	0.88	0.43
**2OW3**	2.958×10^−69^	**1.841×10** ^−^ **^76^**	1.25	0.93
**3L1S**	3.348×10^−18^	**6.093×10** ^−^ **^72^**	0.69	0.58
**4B7T**	0.172	**1.388×10** ^−^ **^33^**	1.45	1.06
**4J71**	0.019	**3.203×10** ^−^ **^22^**	0.62	0.43
**4NM3**	**1.502×10** ^−^ **^41^**	0.040	1.33	1.14
**4PTG**	3.026×10^−20^	**1.439×10** ^−^ **^30^**	1.65	1.34

Bolded data: better discrimination.

**Figure 2:**
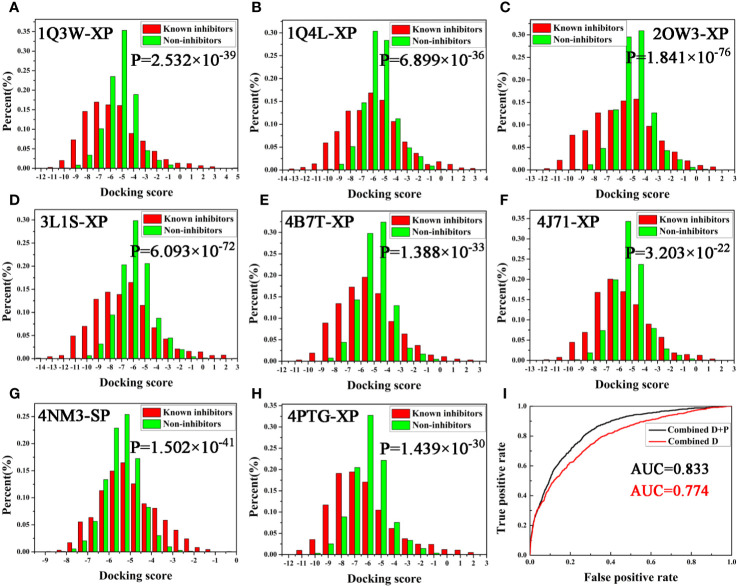
**(A–H)** Eight GSK3β complexes have better ability to distinguish known inhibitors and non-inhibitors under SP or XP docking accuracy; **(I)** AUC value of Bayesian model constructed based on docking score or integrating docking scores and pharmacophore fit values.

Next, the screening power was estimated using the Student’s t-test (P-value), which was conducted to assess the different distributions between the inhibitors and non-inhibitors under SP or XP score modes (Shen et al., 2020). As shown in **Table 1** and **Figure 2**, the molecular docking can effectively distinguish the inhibitors form non-inhibitors, the P-values of eight complexes all far lower than 0.05, except the SP mode of 4B7T. However, discrimination for certain structures still exhibits significant different sampling powers (**Figure 2**). For instance, the P-value of the SP mode for 2OW3 is 2.958 ×10^−69^, while that for 4B7T is only 0.172 (**Table 1**). In general, the XP scoring mode exhibited more accurate determination than the SP scoring mode (**Figure 2**). Therefore, it is necessary to choose an appropriate docking mode for a specific protein structure to ensure docking prediction accuracy. In summary, our molecular docking model based on the eight GSK3β structures with *Glide* could fulfill the requirement of satisfactory docking. Thus, the docking-based VS with the multiple structures would be a reliable tool for the development of potential GSK3β inhibitors.

### Validation of Pharmacophore-Based Virtual Screening

Eight complexes of GSK3β were employed to generate the pharmacophore models, and the models were ranked through the selective scores detected by the GFA algorithm. The results were tabulated in **Table 2**. The prediction capacity of each pharmacophore model to distinguish the inhibitors and non-inhibitors was evaluated with the AUC values, and it indicates that the models contain satisfactory prediction power when AUC > 0.7 (Tian et al., 2013b). According to **Table 2**, feature(S) and feature(D) were analyzed and compared.

**Table 2:** The selectivity scores and AUC values of feature(S) and feature(D).

ID	Feature(S)^a^	Selectivity score	AUC	Feature(D)^a^	Selectivity score	AUC
**1Q4L**	AADHHN	11.701	0.612	ADHN	9.3532	**0.711**
**4NM3**	AADNNN	14.964	0.502	AADNNN	14.964	0.502
**3L1S**	AADHHH	10.447	0.595	AADHH	8.9325	0.657
**2OW3**	ADHHH	8.5895	0.604	DHH	5.5599	**0.709**
**4B7T**	AAHH	5.7496	0.514	AHH	4.2348	0.567
**4PTG**	ADH	5.3541	0.544	AHH	4.4406	0.564
**4J71**	ADH	4.8740	0.601	ADH	4.8740	0.601
**1Q3W**	AAD	5.0798	0.694	AAD	5.0798	0.694

^a^A, hydrogen-bond receptor; D, hydrogen-bond donor; H, hydrophobic; N, negative charge.

Bolded data: better discrimination.

Generally speaking, a pharmacophore model with a higher selectivity score shows a stronger prediction power. But as shown in **Table 2**, we found that most models with the high selectivity score contain a poor discrimination power, such as 4NM3 (AUC < 0.7). Some models, especially the feature(D) of 2OW3, with a low selective score, show a fair inhibitor selectivity on the contrary. It seems that the pharmacophore models using feature(D) are more suitable for our VS protocol. Therefore, to simultaneously avoid a high rate of false positives and find more active GSK3β inhibitors, 1Q4L and 2OW3 models with feature(D) were chosen to perform pharmacophore-based VS (AUC > 0.7, **Table 2**).

### Naive Bayesian Classifiers With Multiple GSK3β Structures

As described above, two protocols based on molecular docking and pharmacophore were respectively optimized for the following VS. In order to further improve the hit rate of screening, a parallel virtual screening based on multiple structures integrating molecular docking and pharmacophore was built and the prediction capabilities were evaluated by NBC model. The flow diagram of the model generation process is revealed in **Figure 1**. The data matrix consists of all the docking scores and fit values of the compounds in the validation dataset for each complex. According to the above results, the docking mode with a higher P-value and/or pharmacophore model with a fair AUC value should be selected as the independent variables to build the NBC models (bolded in **Tables 1** and **2**). Firstly, the prediction power based on the chosen docking score was estimated and the AUC = 0.774 (**Figure 2I**), this value was slightly better than that of the above pharmacophore. But when the model combined the docking scores and fit values, the accuracy was significantly improved, the classifier achieved an AUC of 0.833, indicating that the VS integrating a machine learning based on multi-conformational GSK3β could effectively highlight the active GSK3β inhibitors.

### GSK3β Inhibition Assay

**Figure 3:**
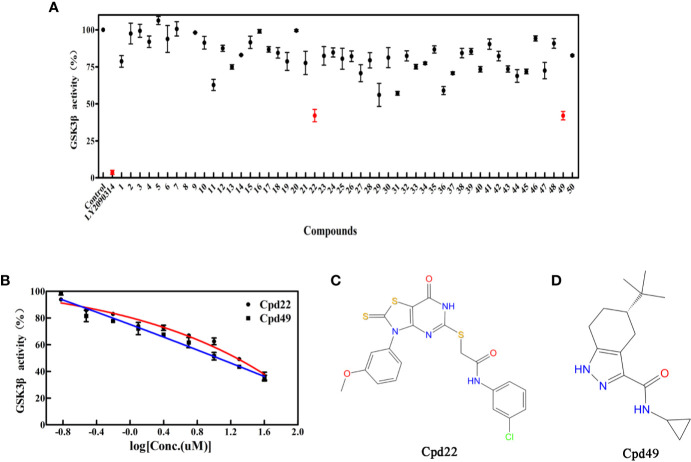
**(A)** Preliminary screening results of 50 compounds with enzyme experiments; **(B)** Cpd22 and Cpd49 concentration gradient results; **(C, D)** 2D structure diagram of Cpd22 and Cpd49.

To determine the GSK3β inhibitory activity of those hit molecules from VS, we performed GSK3β inhibition assays based on ADP-Glo method, in which LY2090314 (Atkinson et al., 2015; Gray et al., 2015; Kunnimalaiyaan et al., 2018) was used as the reference compound. The 50 compounds were tested at the initial concentration of 20 μM. The results of the GSK3β inhibitory activities of these hits are shown in **Figure 3A**. Among the tested compounds, Cpd22 showed effective inhibiting GSK3β activity 57.6% and Cpd49 inhibiting GSK3β activity 58.5% at the concentration of 20 μM, respectively. Afterward, the GSK3β inhibition potency (IC_50_) of Cpd22 and Cpd49 was identified, both compounds could potentially inhibit the activity of GSK3β, with IC_50_ of 15.49 μM and 9.34 μM, respectively (**Figure 3B**). The 2D structures of the two inhibitors are illustrated in **Figures 3C, D**, and both compounds present new scaffolds.

### PAINS Assessment for Cpd49

Cpd49 with the better bioactivity was then investigated whether it could be categorized as a PAINS compound. PAINS refer to a series of promiscuous compounds specifically binding to different macromolecular targets and leading to misleading false-positive results in experimental assays (Baell and Holloway, 2010). The assessment results from Badapple prediction were shown in **Table 3**. The cyclopropane of Cpd49 exhibits a moderate pScore value with 185 and shows a “True” inDrug result. While the tetrahydroindazole group presents a low pScore value with a “False” result inDrug database. Besides, the scaffold3 generated no pScore and the inDrug result is “False”. These results indicate Cpd49 would be a GSK3β selective inhibitor.

**Table 3:** The pScore and inDrug values from Badapple prediction.

Compound	Scaffold Number	Scaffold Structure	pScore^a^	inDrug^b^	
**Cpd49**	1	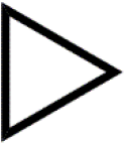	185	True
	2	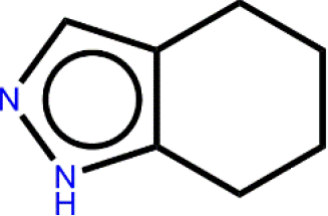	83	False
	3	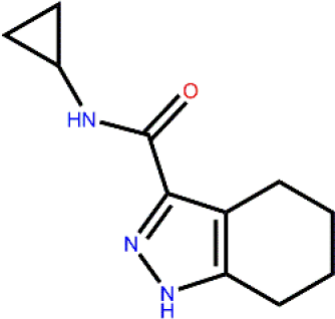	None	False

^a^pScore values, <100 (low), no indication; 100–300 (moderate), weak indication of promiscuity; >300 (high), strong indication of promiscuity.

^b^inDrug, True, means it was found in the drug data base; False, means not found.

### Molecular Docking Analysis

**Figure 4:**
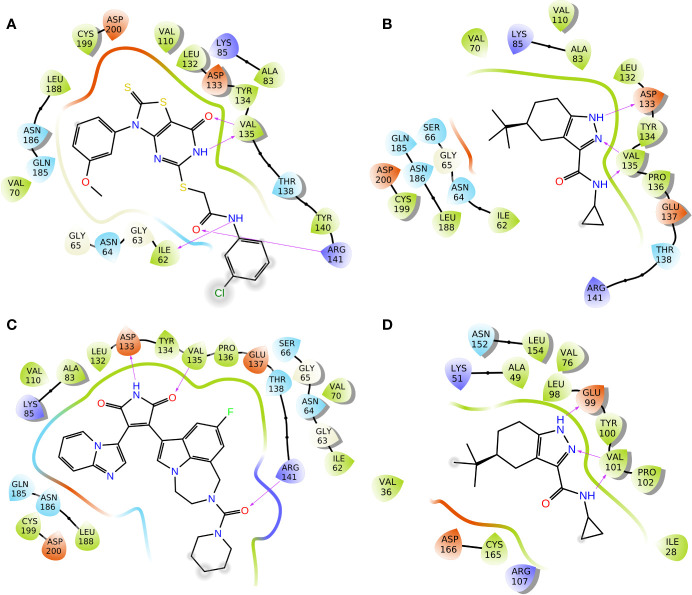
2D presentations of the interactions between GSK3β and **(A)** Cpd22; **(B)** Cpd49; **(C)** LY2090314; **(D)** 2D presentations of the interactions between GSK3α and Cpd49 (H-bonds colored in magenta).

To elucidate the binding mode of inhibition of GSK3β by Cpd22 and Cpd49, the two inhibitors were docked to the crystal structure 1Q4L. The docking scores were −9.032 kcal/mol for GSK3β/Cpd22, and −9.566 kcal/mol for GSK3β/Cpd49. The interactions between the inhibitors and GSK3β have respectively illustrated in **Figure 4**. The docking results indicated that Cpd22 and Cpd49 interact with GSK3β in the same position, in which residues Ile62, Val70, Ala83, Val110, Asp133, Tyr134, Val135, Thr138, Arg141, Leu188, Cys199, and Asp200 constructed the binding pocket and interacted with the inhibitors. Meanwhile, hydrophobic interactions were formed with residues Ile62, Val70, Ala83, Val110, Tyr134, Val135, Leu188, and Cys199 (shown in black lines in **Figures 4A, B**), which was consistent with the analytical results from our previous research. These residues are important to stabilize the binding affinity between the inhibitors and GSK3β (Zhu et al., 2019). In addition, there are several key hydrogen bonds (H-bond) were formed in both complexes. **Figure 4A** showed that Cpd22 formed four H-bonds with Ile62, Val135, and Arg141. For Cpd49, Val135 formed two H-bonds with the pyrazole and nitro group, and Asp133 also formed an H-bond. These H-bond interactions could maintain the specific binding interactions between the ligand and GSK3β (Zhao et al., 2017). Herein, LY2090314, as a positive control, was also docked into the GSK3β protein, and the result was illustrated in **Figure 4C**. Similar to Cpd49, LY2090314 formed the two H-bond interactions with Asp133 and Val135 of GSK3β (**Figure 4B**). Moreover, the carbonyl group of LY2090314 formed an H-bond with Arg141, similarly, the Arg141 of GSK3β also form H-bond with the carbonyl group of Cpd22 (**Figure 4A**). That may be the reason why LY2090314 contains more potent bioactivity than Cpd22 and Cpd49, because of the more H-bond interactions with GSK3β. Finally, Cpd49 was chosen to investigate the interaction with GSK3α, **Figure 4D** shows that the binding pose of Cpd49 in the binding site of GSK3α is almost same with that in GSK3β, it is not surprised because of the high sequence identity between GSK3α and β. Although Cpd49 formed three H-bonds with GSK3α, the docking score, -6.783 kcal/mol, was far lower than GSK3β, indicating that Cpd49 preferred to bind with GSK3β.

### Molecular Dynamic Simulations Analysis

**Figure 5:**
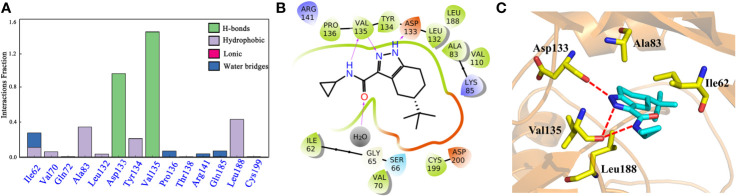
**(A)** the Cpd49-GSK3β residues interaction spectrum; **(B)** 2D presentations of the MD simulation interactions between GSK3β and Cpd49; **(C)** 3D presentations of the MD simulation interactions between GSK3β and Cpd49.

To explore the dynamic binding process of the studied inhibitor, Cpd49 with the best bioactivity was submitted to 200-ns MD. Firstly, the backbone RMSD of the complex was calculated and the result illustrated in **Figure S3**. The plot showed that the system reached equilibrium after 200 ns simulation. In order to understand the mechanism of ligand adaptation in binding space of GSK3β, the per-residue contributions in ligand binding were calculated and the key residues-inhibitor interactions were rendered in **Figure 5A**. As the appears at first glance, several residues formed the strong hydrophobic interactions with Cpd49, including Ile62, Ala83, Tyr134, and Leu188, these residues could form the strong non-polar interactions with GSK3β selective inhibitors, which make the dominating force for the high GSK3β affinity (Zhu et al., 2019), meanwhile, Asp133 and Val135 form strong H-bonds with Cpd49, these hydrogen bond interactions are significantly important for the specific GSK3β binding of the selective inhibitors (**Figure 5B**), which is consistent with the docking analysis discussed above. However, Val135 exhibits more favorable contributions to Cpd49 than Asp133. As shown in **Figure 5C**, the pyrazole group of Cpd49 was closer to Val135 and then formed two H-bonds with Val135. it can be perceived Val135 is a critical residue for selective binding to GSK3β. As shown in **Figure 5C**, Cpd49 was sandwiched between residues Ala83 and Leu188, and encompassed by residues Ala83 above and residues Leu188 below, that formed strong non-polar interactions which highly improved the binding affinity to GSK3β for Cpd49. Overall, the above results prove that Asp133, Val135, Ala83, and Leu188 may be the key residues for inhibitor binding.

**Figure 6:**
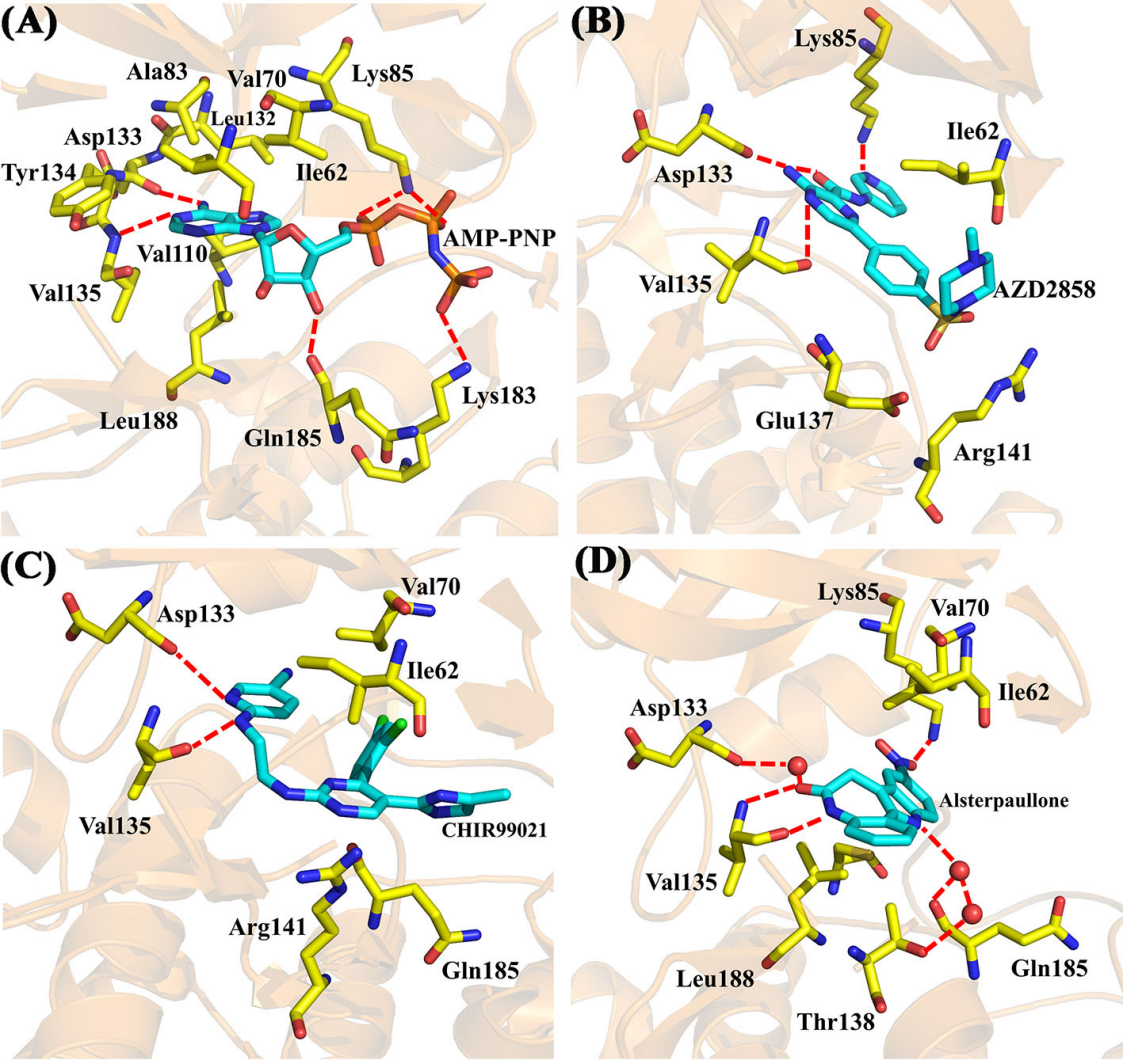
X-ray crystal structures of **(A)** GSK3β/AMP-PNP (PDB ID 1PYX); **(B)** GSK3β/AZD2858 (PDB ID 4ACD); **(C)** GSK3β/CHIR-99021 (PDB ID 5HLN); **(D)** GSK3β/Alsterpaullone (PDB ID 1Q3W).

Moreover, four X-ray crystallographic structures of GSK3β complexes, including AMP-PNP (PDB ID 1PYX) (Bertrand et al., 2003) and three inhibitors under clinical investigation (Xu et al., 2019), Alsterpaullone (PDB ID 1Q3W) (Bertrand et al., 2003), AZD2858 (PDB ID 4ACD) (Berg et al., 2012), and CHIR-99021 (PDB ID 5HLN) (Wagner et al., 2016), were chosen to compare to the binding configuration of Cpd49/GSK3β complex. The crystal structure of GSK3β/AMP-PNP was illustrated in **Figure 6A**. The adenine of AMP-PNP forms two H-bonds with Asp133 and Val135, respectively. Asp133 and Val135 are two key hinge residues, which plays a critical role in the GSK3β specific binding for a selective inhibitor (Pandey and DeGrado, 2016). As shown in **Figure 5C**, the tetrahydroindazole group of Cpd49 could mimic the adenine of ATP to form the key H-bonds with Asp133 and Val135. Similar H-bond interactions occur in AZD2858 and CHIR-99021 systems (**Figures 6B, C**). Besides, the oxygen atom of the ribose could hydrogen bond to Gln185, and the phosphate group of AMP-PNP could form the H-bonds with Lys85 and Lys183, respectively **(** **Figure 6A**) (Bertrand et al., 2003). The H-bond with Lys85 is also formed in AZD2858 system, which is caused by the internal hydrogen bond on the pyrazine ring (**Figure 6B**) (Berg et al., 2012), while these interactions all lose in Cpd49/GSK3β system, which may result in lower bioactivity of Cpd49. On the other hand, the hydrophobic interaction is important for the GSK3β affinity. In AMP-PNP system, these residues consist of Ile62, Val70, Ala83, Val110, Leu132, Tyr134, and Leu188 (Bertrand et al., 2003), and Cpd49 could form the similar non-polar interactions, especially with Ile62, Ala83, Tyr134, and Leu188 (**Figure 5A**). The binding mode of Alsterpaullone/GSK3β shows in **Figure 6D**. First, Alsterpaullone forms two H-bonds with Val135, but unlike Cpd49 system, Alsterpaullone forms a water bridge with Asp133, and the other two water bridges also form between Alsterpaullone and Thr138/Gln185 (Bertrand et al., 2003). Similar to AMP-PNP and AZD2858, Alsterpaullone also could form a strong H-bond with Lys85 (**Figure 6D**). Second, Alsterpaullone also forms strong van der Waals interactions with GSK3β, especially Ile62 and Val70, so is in the CHIR-99021 system (**Figure 6C**) (Wagner et al., 2016), while Cpd49 loses that interaction with Val70 (**Figure 5A**). Thus, all these extra interactions discussed above may lead to the higher GSK3β binding affinity for Alsterpaullone than Cpd49

## Conclusion

In the present study, a parallel VS strategy based on multiple GSK3β protein structures was developed to screen against a large chemical library, in which the NBC model combining molecular docking and pharmacophore show a reliable prediction capability. After a series of biochemical studies, two GSK3β inhibitor hits (Cpd22 and Cpd49) were identified from 50 virtual screened compounds, that highlights the high prediction accuracy and the robust reliability of the integrated machine learning-based VS. Besides, the binding modes between GSK3β and two inhibitors were identified by molecular docking, and some key residues critical to GSK3β selectivity were highlighted through MD simulation. We hope that this study would provide some guidance for the virtual screening or design of novel GSK3β inhibitors.

## Data Availability Statement

The data that support the findings of this study are available from the corresponding author upon reasonable request.

## Author Contributions

JZ and JJ developed the study concept and design. YW and KL performed the modeling studies and carried out the data analysis. MW performed the bio-assay evaluation. LX provided software. JZ, YW, and YaC drafted the manuscript. YuC and JJ approved the manuscript.

## Funding

The study was supported by the National Natural Science Foundation of China (No. 21807049 and 81803430), the Fundamental Research Funds for the Central Universities (JUSRP51703A), the Postgraduate Research & Practice Innovation Program of Jiangsu Province (KYCX19_1888), and the Natural Science Foundation of Jiangsu Province (No. BE2019650).

## Conflict of Interest

The authors declare that the research was conducted in the absence of any commercial or financial relationships that could be construed as a potential conflict of interest.

The reviewer HS declared a past co-authorship with one of the authors, LX, to the handling editor.
