# Supplementary material for: Integrating Machine Learning-Based Virtual Screening With Multiple Protein Structures and Bio-Assay Evaluation for Discovery of Novel GSK3β Inhibitors
Source: Front Pharmacol. 2020 Sep 11;11:566058. doi: 10.3389/fphar.2020.566058 (PMC7517831; doi:10.3389/fphar.2020.566058)
Supplement: Supplementary file 1 [file DataSheet_1.docx]

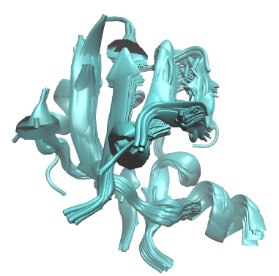


**Figure S1.** The alignment of all GSK3β complexes


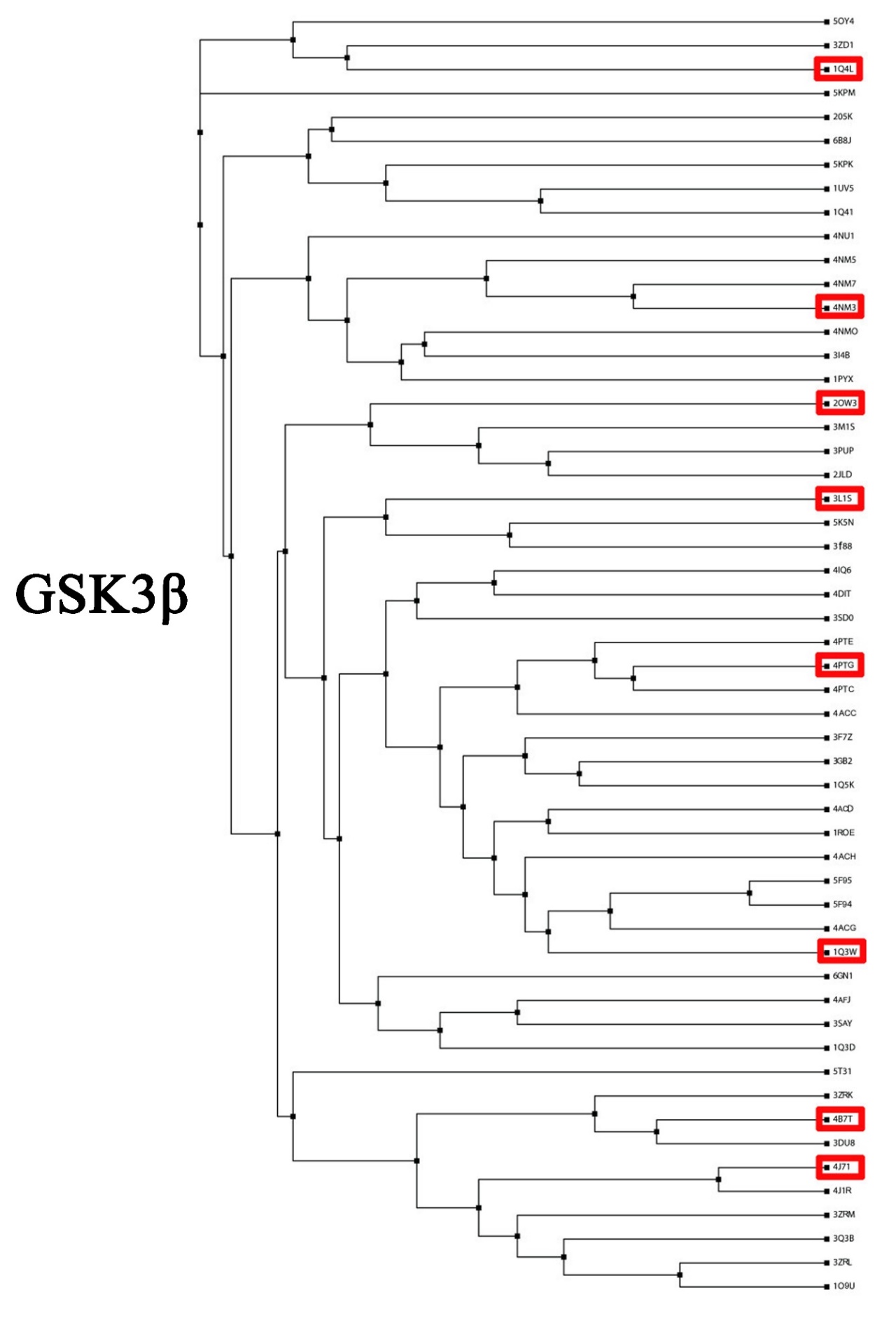


**Figure S2.** Structural clustering for the crystal structures of GSK3β by using the *phylogenetic tree* method in VMD, the eight selected compounds are highlighted in red


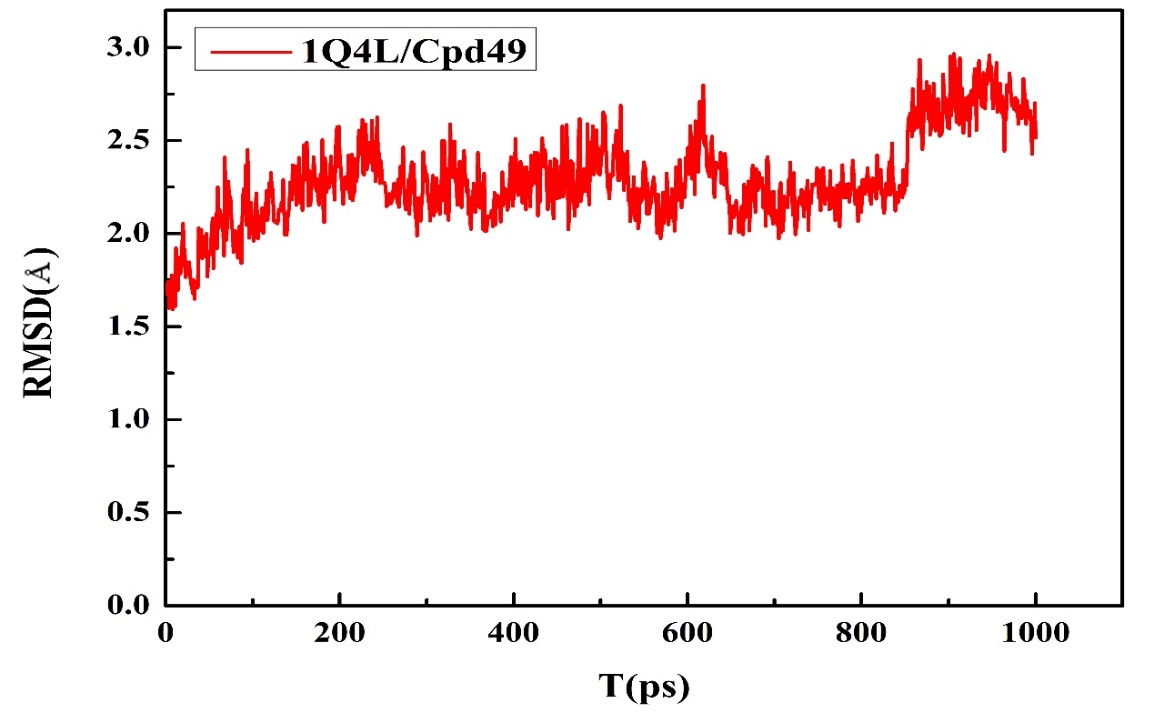


**Figure S3.** The root-mean-square deviation (RMSD) of the GSK3β/Cpd49 complex
